# CD4^+^ T-Cell-Independent Secondary Immune Responses to *Pneumocystis* Pneumonia

**DOI:** 10.3389/fimmu.2016.00178

**Published:** 2016-05-11

**Authors:** Nicholas M. de la Rua, Derrick R. Samuelson, Tysheena P. Charles, David A. Welsh, Judd E. Shellito

**Affiliations:** ^1^Section of Pulmonary/Critical Care and Allergy/Immunology, Department of Medicine, Louisiana State University Health Sciences Center, New Orleans, LA, USA

**Keywords:** immune memory, pneumonia, *Pneumocystis*, CD8^+^ T-cells, macrophages, BMPCs

## Abstract

*Pneumocystis* pneumonia is a major cause of morbidity and mortality among immunocompromised patients, especially in the context of HIV/AIDS. In the murine model of *Pneumocystis* pneumonia, CD4^+^ T-cells are required for clearance of a primary infection of *Pneumocystis*, but not the memory recall response. We hypothesized that the memory recall response in the absence of CD4^+^ T-cells is mediated by a robust memory humoral response, CD8^+^ T-cells, and IgG-mediated phagocytosis by alveolar macrophages. To investigate the role of CD8^+^ T-cells and alveolar macrophages in the immune memory response to *Pneumocystis*, mice previously challenged with *Pneumocystis* were depleted of CD8^+^ T-cells or alveolar macrophages prior to re-infection. Mice depleted of CD4^+^ T-cells prior to secondary challenge cleared *Pneumocystis* infection within 48 h identical to immunocompetent mice during a secondary memory recall response. However, loss of CD8^+^ T-cells or macrophages prior to the memory recall response significantly impaired *Pneumocystis* clearance. Specifically, mice depleted of CD8^+^ T-cells or alveolar macrophages had significantly higher fungal burden in the lungs. Furthermore, loss of alveolar macrophages significantly skewed the lung CD8^+^ T-cell response toward a terminally differentiated effector memory population and increased the percentage of IFN-γ^+^ CD8^+^ T-cells. Finally, *Pneumocystis*-infected animals produced significantly more bone marrow plasma cells and *Pneumocystis*-specific IgG significantly increased macrophage-mediated killing of *Pneumocystis in vitro*. These data suggest that secondary immune memory responses to *Pneumocystis* are mediated, in part, by CD8^+^ T-cells, alveolar macrophages, and the production of *Pneumocystis-s*pecific IgG.

## Introduction

Pneumonia due to the opportunistic human fungal pathogen *Pneumocystis jirovecii* is an AIDS-defining illness, and there is a direct inverse relationship between CD4^+^ T-cell counts in the blood and the risk for infection (1). *Pneumocystis* is also a major cause of mortality in patients whose CD4^+^ T-cell number or function is significantly depressed due to malignancy, chemotherapy, or other immunosuppression (1, 2). Animal models of immunodeficiency demonstrate that the loss of CD4^+^ T-cells renders mammals susceptible to *Pneumocystis* lung infection (2). Additionally, CD4^+^ T-cells from *Pneumocystis*-infected mice are able to mediate clearance of *Pneumocystis* infection upon adoptive transfer into Rag1^(−/−)^ mice (3). As such, CD4^+^ T-cells have been a primary focus in the study of host defense against this pathogen.

However, it has also been shown that adoptive transfer of hyperimmune serum mediates effective passive immunity to *Pneumocystis* in the absence of T cells (4). Further, Gigliotti and colleagues have shown that immunocompetent mice immunized against *Pneumocystis* by intratracheal inoculations with *Pneumocystis* are protected from subsequent lung infection following depletion of CD4^+^ T cells with anti-CD4 monoclonal antibodies, which suggests that CD4^+^ T-cells are not required for the memory recall response (5). In addition, these investigators demonstrated that antibodies are responsible for the observed protection against *P. carinii* in the absence of CD4^+^ T cells (5). These results suggest that CD4^+^ T-cells are not required for secondary immune recall to *Pneumocystis* infection. These results also suggest that more focus should be given to immune cells other than CD4^+^ T-cells in host defense against *Pneumocystis*. This is highlighted by the fact that exposure to *Pneumocystis* is ubiquitous in humans and that the memory response to infection is often called into play in the context of CD4^+^ T-cell depletion (HIV infection).

In this study, we evaluated the cellular immune memory recall response to murine *Pneumocystis* infection in the absence of CD4^+^ T-cells. Specifically, we depleted CD8^+^ T-cells or alveolar macrophages prior to a secondary infection and evaluated the host’s memory response to *Pneumocystis* infection. Mice depleted of CD4^+^ T-cells prior to secondary challenge cleared *Pneumocystis* infection within 48 h identical to immunocompetent mice during a secondary memory recall response. However, loss of CD8^+^ T-cells or macrophages prior to the memory recall response significantly impaired *Pneumocystis* clearance. Specifically, mice depleted of CD8^+^ T-cells or alveolar macrophages had significantly higher fungal burden in the lungs, and loss of alveolar macrophages significantly increased the percentage of IFN-γ^+^ CD8^+^ T-cells. Finally, *Pneumocystis*-infected animals generated significantly more bone marrow plasma cells, and *Pneumocystis*-specific IgG purified from sera of previously infected mice significantly increased macrophage-mediated killing of *Pneumocystis in vitro*. These data suggest that secondary immune memory responses to *Pneumocystis* are mediated, in part, by CD8^+^ T-cells and alveolar macrophages, in addition to the production of *Pneumocystis*-*s*pecific IgG.

## Materials and Methods

### Immune Memory Model

*Pneumocystis murina* was propagated in B10;B6 Rag2/IL2rg double knockout mice from Taconic (Model 4111F; Hudson, NY, USA). Inocula and naive lung homogenates were prepared as previously described (2), and recipient mice were infected, as previously described (6). C57Bl/6 female mice (6–8 weeks) were either intratracheally challenged with 2 × 10^5^ cysts or naive lung homogenate. Mice were depleted of CD4^+^ T-cells, CD8^+^ T-cells, or macrophages at the indicated time points prior to infection by intraperitoneal (i.p.) injection with 100 μg of anti-CD4 mAb (hybridoma GK 1.5; Taconic), 100 μg of anti-CD8 mAb (hybridoma 58.6.72; National Cell Culture Center), or intratracheal administration (i.t.) of 100 μL of clodronate liposomes (http://clodronateliposomes.com; Netherlands). Control animals were given IgG isotype control *via* i.p. injection or PBS containing empty liposomes *via* i.t. inoculation. Depletions were maintained by dosing animals every 6 days, which is sufficient to maintain significant cellular depletion (data not shown).

### Quantitation of *Pneumocystis*-Specific IgG

Whole blood was either collected from tail bleeds or cardiac puncture, and serum was obtained *via* centrifugation of whole blood in BD serum separator tubes at 1,500 × *g* for 10 min at 4°C. Serum was stored at −20°C. *Pneumocystis* whole cell lysate was used to quantify the IgG humoral response. In brief, *Pneumocystis*-infected lungs from B10;B6 Rag2/IL2rg double knockout mice were homogenized over a 70-μm filter and washed with chilled magnesium-free PBS supplemented with 5% glutathione. The filtrate was centrifuged at 300 × *g* for 10 min, and the pellet was resuspended in approximately 1 mL of residual supernatant. The resuspended pellet was then pipetted on top of 30 mL of 1:2 Centricoll (density ~1.40; Sigma C-0580) diluted in PBS. The preparation was centrifuged for 15 min at 275 × *g*. *Pneumocystis* organisms were collected from the PBS–Centricoll interphase and were washed with PBS containing 5% glutathione. The pellet was then sonicated using a water bath sonicator. Total protein was quantified using Nanodrop spectrophotometer. Total *Pneumocystis*-specific IgG was quantified using Mouse IgG Total ELISA Ready-SET-Go Kit (Affymetrix eBiosciences). *Pneumocystis* whole cell lysate was coated on a Thermo Scientific Nunc 96-well flat bottom plates at 5 μg/mL in 50 μL of 0.1M Na_2_CO_3_/0.1M NaHCO_3_ (pH 9.5) per well. The plates were incubated overnight at 4°C. Wells were aspirated and washed twice with PBS 0.05% Tween20. Plates were blocked according to the manufacture’s protocol. Serum was diluted 1:500 and allowed to incubate in the presence of anti-mouse IgG HRP-conjugated detection antibodies for 3 h at room temperature with shaking at 400 rpm. Plates were aspirated and washed four times. Plates were developed with TMB solution for 15 min at room temperature; the reactions were stopped *via* the addition of 2 N H_2_SO_4_. Optical density (OD) was recorded at 450 nm with a BioTek ELX800 plate reader. Values are reported as the mean OD450 ± SEM with the value of the control wells (BSA coated) subtracted.

### Quantitation of *Pneumocystis* Fungal Burden

The right lung was placed in 1 mL of Trizol and briefly stored at −20°C. RNA was extracted from each lung following the manufacturer’s protocol. RNA was quantified using a Nanodrop spectrophotometer. cDNA was synthesized from 1 μg of Lung RNA using BioRad Reverse Transcription Supermix for RT-qPCR following the manufacturer’s protocol. *Pneumocystis* fungal burden was assayed *via* amplifying a 166-bp fragment of the small ribosomal rRNA (ssRNA) from the mitochondrial genome, as described previously (6, 7).

### Flow Cytometric Analysis of T-Cells, B-Cells, and Macrophages in the Lung

Left lung tissue was homogenized over a 70-μm filter with 10 mL of homogenization buffer that comprised RPMI 1640 10% FBS, 1 mg/mL collagenase type 1 (Worthington Biochemical, Lakewood, NJ, USA), and 30 μg/mL DNAse I (Roche Diagnostics, Indianapolis, IN, USA). Lung homogenates were incubated at 37°C with shaking at 200 rpm for 30 min. All cell suspensions were centrifuged at 300 × *g* for 10 min at 4°C. Cells were resuspended in 5 mL of chilled 1× RBC lysis buffer (Biolegend, San Diego, CA, USA) for 4 min on ice. RBC lysis reactions were diluted with 25-mL chilled PBS. Lymphocytes were enumerated using a hemocytometer and trypan blue exclusion. Prior to all extracellular staining, cells were treated with TruStain fcX (Biolegend) to block Fc receptors. To conduct extracellular phenotype studies, 1 × 10^6^ cells were stained with various combinations of the following fluorochrome-conjugated Abs specific for murine CD3, CD4, CD8, CD19, CD28, CD45, CD45RA, CD197 (CCR7), CD11b, CD11c, and Zombie NIR (Biolegend) suspended in MACS buffer (Miltenyi Biotec, San Diego, CA, USA) at the manufacturer’s recommended concentrations for 30 min at 4°C.

Pan T-cell, B-cells, and macrophages in the lung were assayed by gating singlet events (FSH vs. FSA), lymphocyte size gate (FSC vs. SSC), and selecting viable CD45^+^ events indicated by a CD45 expression and lack of uptake of Zombie NIR, intracellular uptake of this dye indicates cell death. T-cells were enumerated as a percent of CD45^+^ live events by gating on CD3^+^, CD4^+^, and CD8a^+^ expression. B-cells were enumerated as a percentage of CD45^+^ live events by gating on CD45^+^CD19^+^ expression. Alveolar macrophages were enumerated as a percentage of CD45^+^ live events by gating on CD11c^+^CD11b^−^ events. These data provided evidence of successful depletion and of cellular response to secondary infection with *Pneumocystis*.

In order to assess the CD8^+^ T-cell response to secondary infection with *Pneumocystis*, four extracellular phenotypes for CD8^+^ T-cells were defined as naive (CD45^+^CD3^+^CD4^−^CD8^+^CD45RA^+^CD28^+^), central memory (CD45^+^CD3^+^CD4^−^CD8^+^CD45RA^−^CD197^+^), effector memory (CD45^+^CD3^+^CD4^−^CD8^+^CD45RA^−^CD197^−^), and terminally differentiated effector memory re-expressing CD45RA (CD45^+^CD3^+^CD4^−^CD8^+^CD45RA^+^CD28^−^) cells.

For intracellular staining (ICS) experiments, cells were resuspended in BD GolgiPlug and incubated for 5 h. Following GolgiPlug treatment, cells were stained with TruStain fcX (Biolegends) for 5 min prior to extracellular staining with CD45, CD3, CD4, and CD8 antibodies. Cells were then washed with FACS buffer, and resuspended in Cytofix/Cytoperm (Fixation/Permeablization Kit; BD Biosciences) and incubated for 20 min, as per the manufacturer’s instructions. Cells were then resuspended in 1× Permwash and incubated for 5 min at room temperature. After washing, cells were incubated for 30 min at 4°C with fluorescently labeled anti-mouse IFN-γ, IL-4, IL-17, or GM-CSF antibodies (Biolegend, San Diego, CA, USA), diluted in Permwash at appropriate concentrations. Wells were then washed with FACS buffer and fixed with PBS + 1% formalin. For all experiments, cells were acquired using LSRII flow cytometer (BD Biosciences, San Jose, CA, USA). All analyses of FACS data were performed using FlowJo software Version 9.4 (Tree Star; Ashland, OR, USA). Intracellular phenotyping is presented as a percentage of CD45^+^, CD3^+^CD4^+^, or CD45^+^CD3^+^CD8^+^ events.

### Isolation and Culture of Bone Marrow Plasma Cells to Assess Their Generation after Infection and Potential Secretion of *Pneumocystis*-Specific IgG

In order to assess whether infection with *Pneumocystis* generates *Pneumocystis*-specific bone marrow plasma cells (BMPCs), BMPCs were enumerated using flow cytometry and cultured. BMPCs (CD45R^−^CD138^+^) are long-lived cells that secrete neutralizing antibody after exposure to antigen. As terminally differentiated B-cells of the bone marrow compartment, they do not survive or secrete antibody *in vitro* without the support of mesenchymal stem cells (MSCs). Mouse MSCs were isolated from the femur and the tibia of uninfected animals, as described previously (8). Specifically, the epiphysis from each bone was removed and the bone marrow from the diaphysis was flushed with a 26-gage needle with a syringe filled with culture media, making sure that the bone was flushed completely. Bones were then cut into small chips ~1–3 mm in size and treated with DMEM containing 20% FBS and collagenase II (1 mg/mL) for 1 h at 37°C with shaking at 200 rpm. The bone chips were then resuspended in 10 mL of DMEM containing 20% FBS and 0.1% penicillin–streptomycin for 5 days in a T-25 flask and monitored for mesenchymal cell migration from bone chips and growth onto the flask. After sufficient migration from bone chips (~5–7 days), cells were passed into a T75 flask and maintained in culture for the rest of the experiment.

In order to quantify total BMPCs, mouse BMPCs were enriched from total bone marrow cells *via* the mouse CD138^+^ Plasma Cell Isolation Kit (Miltenyi Biotec). Briefly, cells were resuspended in MACS buffer and incubated with 100 μL of CD138 Microbeads per 10^8^ total cells for 15 min at 4–8°C. Cells were then washed and resuspended in 500 μL of MACS buffer. Plasma cells were then obtained by placing a MS column (Miltenyi Biotec) in the magnetic field of an octoMACs separator (Miltenyi Biotec) and applying the labeled cells to the column and washing two times with MACS buffer. Plasma cells were collected by removing the column from the magnetic field and eluting with MACs buffer. BMPCs were further purified *via* flow cytometric sorting. Briefly, purified BMPCs were incubated Fc block prior to staining with anti-mouse CD138 and anti-mouse/human CD45R/B220 for 30 min at 4°C. Cells were then washed and resuspended in MACS buffer for flow sorting. Enriched BMPCs (CD138^+^/CD45R^−^) were sorted directly into culture media and immediately cultured. Magnetically and flow cytometric-sorted CD138^+^ BMPCs were immediately cocultured with 5 × 10^3^ MSCs in 96-well plates in DMEM containing 20% FBS and 0.1% pen–strep. After 72 h, supernatant was collected from wells containing MSCs, BMPCs, or cocultured BMPCs and MSCs, and *Pneumocystis*-specific IgG was quantified as described above.

### Purification of *Pneumocystis*-Specific Mouse IgG

Sera from animals previously infected with *Pneumocystis* or sera from uninfected animals were pooled and desalted using the Zeba desalting columns (Life Technologies), as per the manufacturer’s instructions. Desalted sera were then used to purify IgG using the Melon Gel Purification Kit (Life Technologies Corporation, Grand Island, NY, USA) as per the manufacturer’s instructions. Purified IgG was then quantified using a Nanodrop spectrophotometer and used for *in vitro* macrophage killing assays.

### Macrophage-Mediated *Pneumocystis* Killing Assay

MH-S alveolar macrophages were acquired from ATTC (ATCC^®^ CRL-2019™). MH-S cells were maintained in complete media (RPMI 1640 containing 0.05-mM 2-mercaptoethanol and 10% FBS) and were passed every 2–3 days throughout the experiment. Macrophage-mediated *Pneumocystis* killing assays were performed by plating 100,000 *Pneumocystis* cysts and 100,000 alveolar macrophages (1:1 ratio) per well in a 96-well flat bottom plate. Each experimental group was run in sextuplet. Purified immune IgG or naive IgG was then added to the wells at the following concentrations (0.002, 0.004, and 0.008 μg), and cultures were incubated for 24 h. Control wells contained cysts + macrophages without the addition of IgG. Following incubation, 250 μL of Trizol was added to each well and incubated for 5 min and repeated one more time. RNA extractions and *Pneumocystis* enumeration were then performed as described above.

### Statistical Analysis

Results are presented as mean ± SEM. Statistical analyses were performed using GraphPad Prism 5 (La Jolla, CA, USA), and statistical significance was measured at *P* ≤ 0.05. The non-parametric Kruskal–Wallis one-way analysis of variance (ANOVA), followed by *post hoc* Dunn’s multiple comparisons of the means, was used for all *in vivo* assays.

## Results

### Immune Memory Recall Does Not Require CD4^+^ T-Cells

To evaluate the immune memory recall responses to *Pneumocystis* pneumonia, we first investigated the requirement of CD4^+^ T-cells during secondary immune responses, as well as establishment of cellular and humoral immune memory. C57BL/6 mice were grouped into the following categories: uninfected animals (designated none), immune-intact animals (isotype), and three groups of mice depleted of CD4^+^ T cells. CD4 depletion was maintained: (a) prior to primary infection and continuously throughout the experiment (cont.), (b) only prior to a primary infection (1°), or (c) only prior to a secondary infection (2°). Mice were infected intratracheally with live *P. murina* (~2 × 10^5^ cysts in 100 μL of PBS) and were allowed to clear the infection naturally (isotype, 1° and 2°) or were given Bactrim (trimethoprim and sulfamethoxazole) *ad libitum* in the water (cont.), following 4 weeks of persistent infection. All mice had no detectable *P. murina* in lung tissue by qPCR prior to re-infection (not shown). Animals were then re-infected with *P. murina* (~2 × 10^5^ cysts in 100 μL of PBS) and pathogen clearance, *P. murina*-specific IgG, and lung immune responses were evaluated.

**Figure 1:**
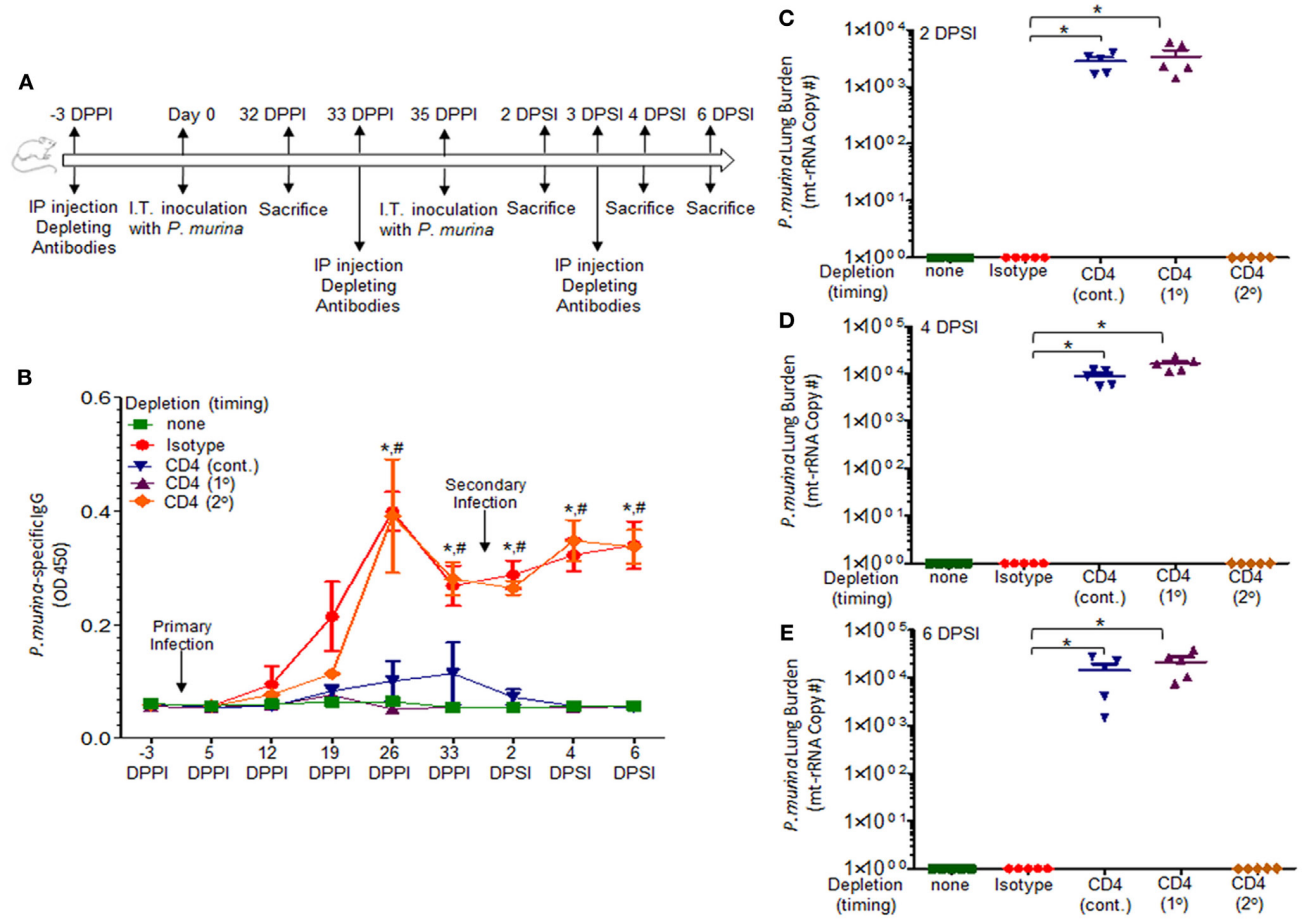

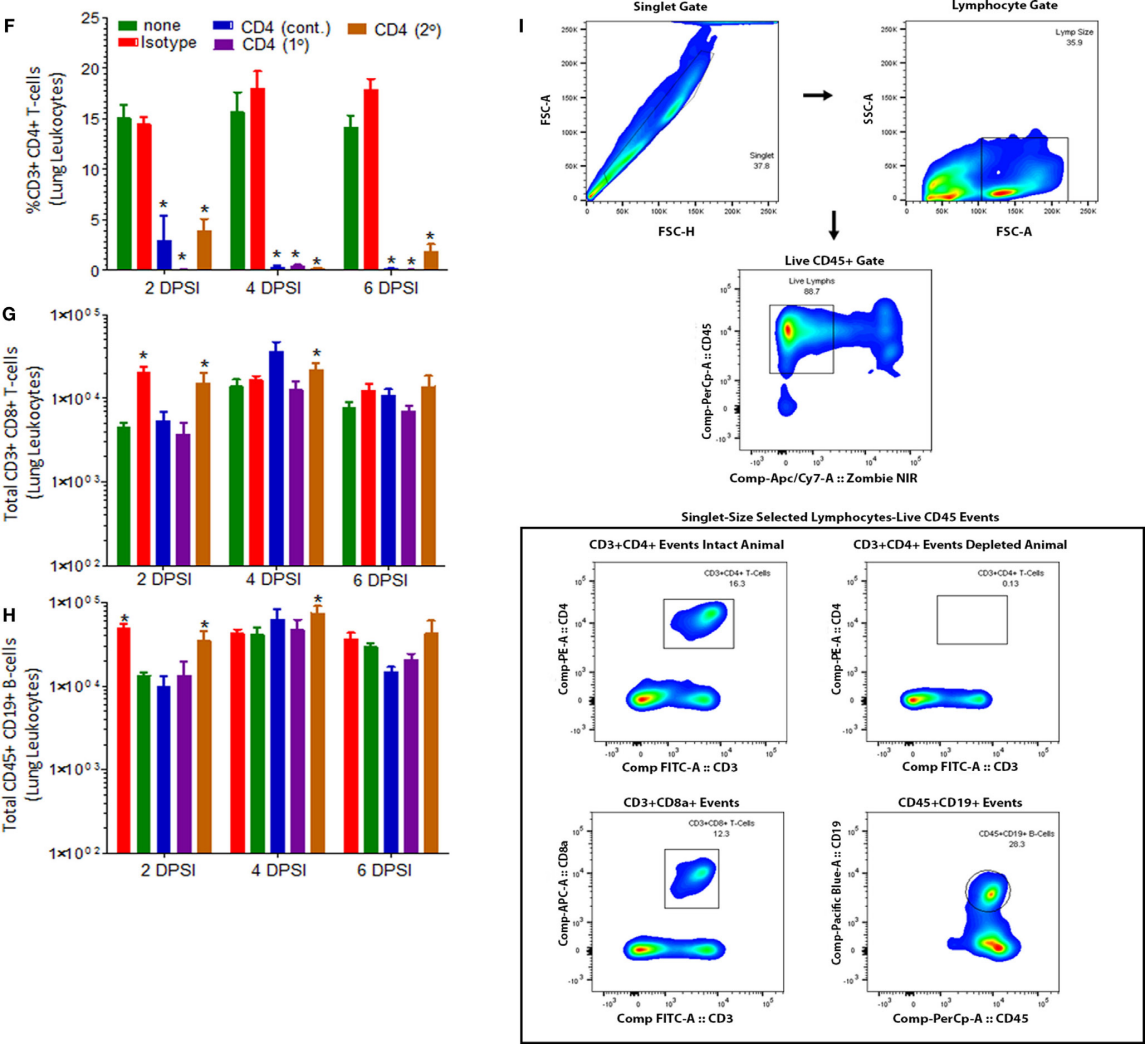
**Immune memory recall does not require CD4^+^ T-cells**. **(A)** Schematic outline of the experimental protocol used in this study. C57BL/6 mice were infected intratracheally with live *P. murina* (~2 × 10^5^ cysts in 100 μL of PBS). Three groups of mice were depleted of CD4^+^ T cells every 6 days with CD4^+^ T-cell depletion occurring continuously throughout the experiment (cont.) or prior to primary infection (1°) or prior to secondary infection (2°). **(B)** *P. murina*-specific IgG in the serum was assayed by ELISA. Levels of *P. murina* in the lung following secondary infection at the following time points: **(C)** 2 days post-lung infection, **(D)** 4 days post-lung infection, and **(E)** 6 days post-lung infection *via* qPCR. Lung immunological responses: **(F)** CD4^+^ T cells, **(G)** CD8^+^ T cells, and **(H)** CD19^+^ B cells were quantified *via* flow cytometry. **(I)** Representative gating strategy used for flow cytometry analysis. Dots and bars represent mean ± SEM, *N* = 5 at each time points, * indicates *P* < 0.05, when comparing isotype to sham-infected animals and ^#^ indicates *P* < 0.05, when comparing CD4 (2°) to sham-infected animals, for each of the indicated time point, by Kruskal–Wallis ANOVA followed by Dunn’s posttest. DPPI, days post-primary infection; DPSI, days post-secondary infection.

Figure 1A describes the experimental design used in this study. *P. murina*-specific IgG in the serum was assayed by ELISA (Figure 1B). Levels of *P. murina* in the lung following secondary infection at the following time points: 2 days post-lung infection (Figure 1C), 4 days post-lung infection (Figure 1D), and 6 days post-lung infection (Figure 1E) were assessed *via* qPCR. We also assessed the levels of CD4^+^ T cells (Figure 1F), CD8^+^ T cells (Figure 1G), and CD19^+^ B cells (Figure 1H) in the lungs using flow cytometry. The representative gating strategy to determine the number of CD4^+^ T cells, CD8^+^ T cells, and CD19^+^ B cells is shown in Figure 1I. We found that CD4^+^ T-cells are required to generate *Pneumocystis*-specific IgG, but are not required for IgG recall responses during re-infection (Figure 1B). Secondary immune memory responses do not require CD4^+^ T-cells, as the levels of *P. murina* in the lung did not significantly differ from immune-intact animals (Figures 1C–E). However, CD4^+^ T-cells are required for generation of immune memory, as mice depleted of CD4^+^ T-cells prior to a primary infection or continuously depleted are unable to mount an effective memory immune response (Figures 1C–E). In fact, mice continuously depleted of CD4^+^ T-cells do not generate any immune memory, as indicated by fungal burden and IgG levels identical to CD4-depleted animals experiencing *P. murina* infection for the first time. In addition, evaluation of the immune cell populations in the lungs following secondary infection showed a significant increase in the number of CD8^+^ T-cells and CD19^+^ B-cells in animals depleted of CD4^+^ T-cells prior to a secondary infection (Figures 1G,H). Levels of CD8^+^ T-cells and CD19^+^ B-cells in mice depleted of CD4^+^ T-cells prior to a secondary infection were similar to those observed in immune-intact animals, suggesting that CD8^+^ T-cells and CD19^+^ B-cells may be important for a maximal secondary immune response. These results further indicate that secondary immune memory responses do not require CD4^+^ T-cells, but that the development of protective memory does require CD4^+^ T-cells during a primary infection. We then went on to investigate other cellular populations that could be involved in secondary immune memory responses to *Pneumocystis*.

### Maximal Host Defense against Secondary Infection Requires CD8^+^ T-Cells and Alveolar Macrophages

**Figure 2:**
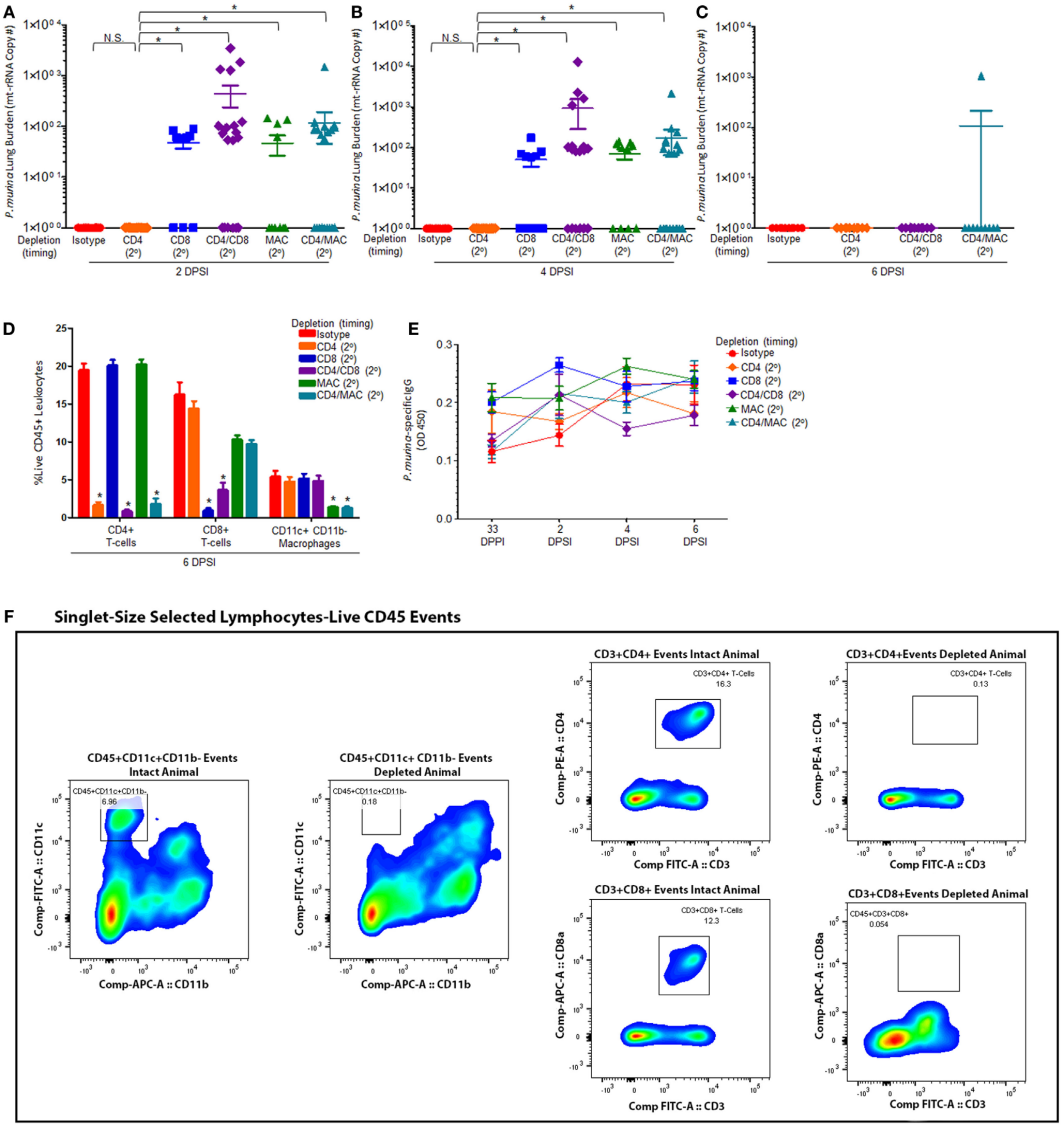
**Maximal immune memory recall requires CD8^+^ T-cells and alveolar macrophages**. C57BL/6 mice were infected intratracheally with live *P. murina* (~2 × 10^5^ cysts in 100 μL of PBS), and groups of mice were depleted of CD4^+^ T cells, CD8^+^ T-cells, CD4^+^ and CD8^+^ T-cells, macrophages, or CD4^+^ T-cells and macrophages every 6 days with depletion occurring prior to secondary infection (2°). Levels of *P. murina* in the lung following secondary infection at the following time points: **(A)** 2 days post-lung infection, **(B)** 4 days post-lung infection, and **(C)** 6 days post-lung infection was assessed *via* qPCR. Lung immunological responses: **(D)** CD4^+^ T cells, CD8^+^ T cells, and CD11c^+^CD11b^−^ macrophages were quantified *via* flow cytometry. **(E)** *P. murina*-specific IgG, in the serum was assayed by ELISA. **(F)** Representative gating strategy used for flow cytometry analysis. Dots and bars represent mean ± SEM, *N* = 10–15 at each time points, * indicates *P* < 0.05, for the indicated comparisons for each of the indicated time point, by Kruskal–Wallis ANOVA followed by Dunn’s posttest. DPPI, days post-primary infection; DPSI, days post-secondary infection.

Mice were infected with *P. murina* as described above, and groups of mice were depleted of CD4^+^ T cells, CD8^+^ T-cells, CD4^+^/CD8^+^ T-cells, macrophages, and CD4^+^ T-cells/macrophages every 6 days with depletion occurring prior to secondary infection (2°). Levels of *P. murina* in the lung following secondary infection at the following time points: 2 days post-lung infection (Figure 2A), 4 days post-lung infection (Figure 2B), and 6 days post-lung infection (Figure 2C) were determined *via* qPCR. Additionally, the levels of lung CD4^+^ T cells (Figure 2D), CD8^+^ T cells (Figure 2D), and CD11c^+^CD11b^−^ macrophages (Figure 2D) were quantified *via* flow cytometry. *P. murina*-specific IgG in the serum was also assessed (Figure 2E). The representative gating strategy to determine the number of CD4^+^ T cells, CD8^+^ T cells, and CD11c^+^CD11b^−^ macrophages is shown in Figure 2F. Flow cytometric analysis demonstrated that all targeted immune cell populations in each group were significantly depleted prior to re-infection (Figures 2D,F). Further, loss of CD4^+^ T cells, CD8^+^ T-cells, macrophages, CD4^+^ and CD8^+^ T cells, or CD4^+^ T cells and macrophages did not significantly affect the levels of *Pneumocystis-*specific IgG after re-challenge, suggesting that the humoral response is still intact (Figure 2E). However, mice depleted of CD8^+^ T-cells prior to secondary infection with *P. murina* had a significant increase in lung burden compared to either CD4-depleted or immune-intact animals at 2 and 4 days post-infection (DPI) (Figures 2A,B), despite similar amounts of *Pneumocystis-*specific IgG when compared to immune-intact animals. Similarly, mice depleted of macrophages exhibited a significantly increased level of *P. murina* in the lungs compared to control mice at 2 and 4 DPI (Figures 2A,B). However, mice depleted of CD8^+^ T-cells or macrophages cleared lung infection at 6 DPI (Figure 2C), suggesting that the cellular recall response is not essential for secondary host defense against *Pneumocystis* infection. These data also suggest that the immune memory response to *Pneumocystis* is, in part, mediated by CD8^+^ T-cells and macrophages, but that CD4^+^ T-cells play little role. We then sought to examine the cellular phenotypes associated with CD4^+^ T-cells and CD8^+^ T-cells during secondary immune responses to *Pneumocystis*.

### Loss of CD8+ T-Cells or Alveolar Macrophages Delays IFN-γ Production by CD4^+^ T-Cells in Secondary Immune Responses to *Pneumocystis*

**Figure 3:**
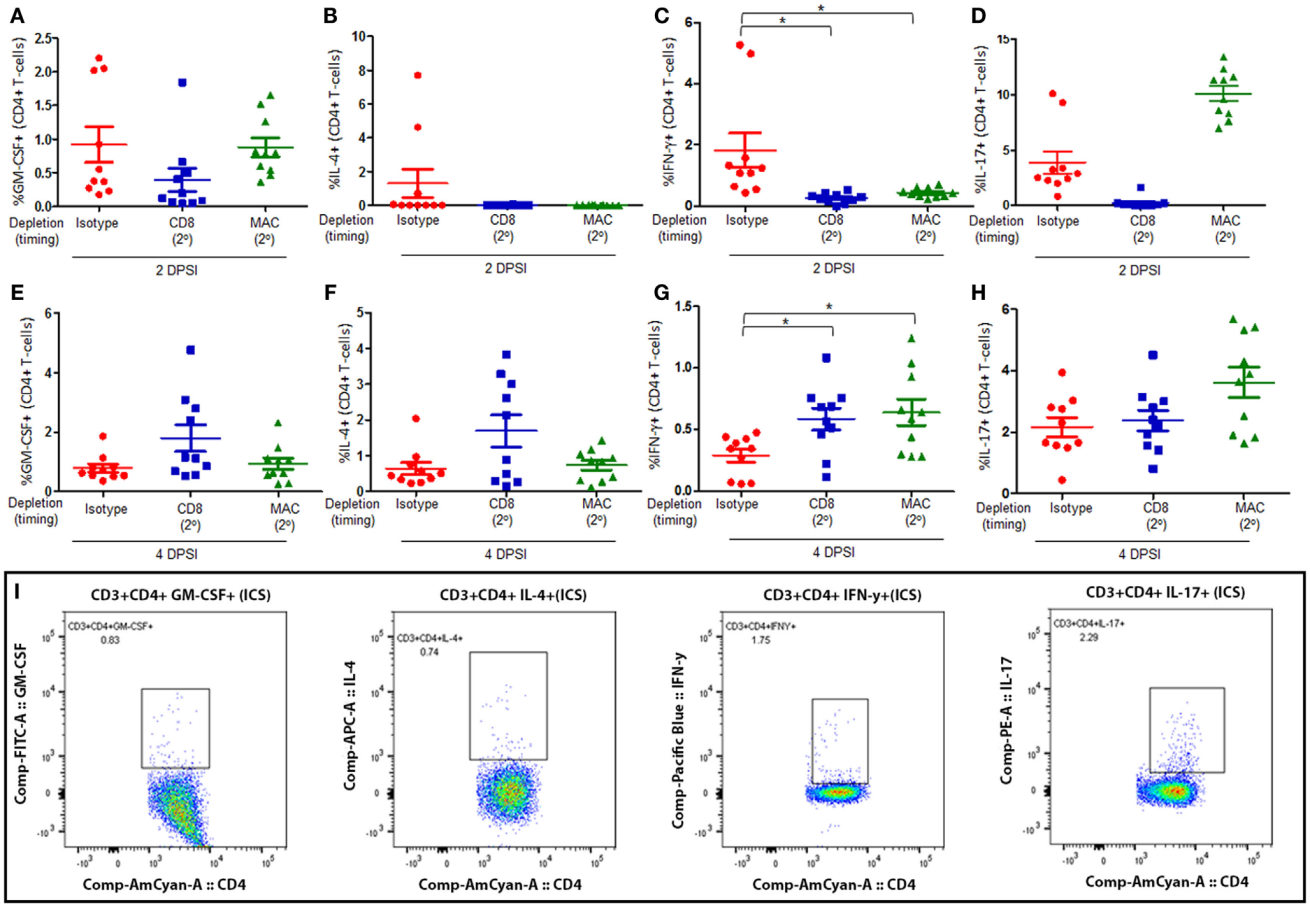
**Loss of CD8**^+^ **T-cells or alveolar macrophages delays IFN-γ production by CD4**^+^ **T-cells in secondary immune responses to *Pneumocystis***. C57BL/6 mice were infected intratracheally with live *P. murina*, and groups of mice were depleted of CD8^+^ T-cells and macrophages every 6 days with depletion occurring prior to secondary infection (2°). Lung CD4^+^ T-cell subsets: GM-CSF^+^CD4^+^ T cells at 2 and 4 days post-secondary infection [**(A,E)**, respectively], IL-4^+^ CD4^+^ T cells at 2 and 4 DPI [**(B,F)**, respectively], IFN-γ^+^CD4^+^ T cells at 2 and 4 DPI [**(C,G)**, respectively], and IL-17^+^CD4^+^ T cells at 2 and 4 DPI [**(D,H)**, respectively], were quantified *via* flow cytometry. **(I)** Representative gating strategy used for flow cytometry analysis. Dots and bars represent mean ± SEM, *N* = 10 at each time points, * indicates *P* < 0.05, for the indicated comparisons for each of the indicated time point, by Kruskal–Wallis ANOVA followed by Dunn’s posttest. DPSI, days post-secondary infection.

Mice were infected with *P. murina* as described above, and groups of mice were depleted of CD8^+^ T-cells and macrophages every 6 days with depletion occurring prior to secondary infection (2°). Lung CD4^+^ T-cell subsets were then examined at 2 and 4 days post-secondary infection: GM-CSF^+^ CD4^+^ T cells (Figures 3A,E, respectively), IL-4^+^ CD4^+^ T cells (Figures 3B,F, respectively), IFN-γ^+^ CD4^+^ T cells (Figures 3C,G, respectively), and IL-17^+^ CD4^+^ T cells (Figures 3D,H, respectively). The gating strategy used to determine the various CD4^+^ T-cell phenotypes are depicted in Figure 3I. The percentage of GM-CSF^+^, IL-17^+^, or IL-4^+^ CD4^+^ T cells in the lung 2 or 4 DPI in immune-intact animals did not significantly differ from CD8-depleted animals or macrophage-depleted mice. Conversely, loss of CD8^+^ T-cells or macrophages significantly decreased the percentage of IFN-γ^+^ CD4^+^ T cells compared to control animals 2 DPI. However, by 4 DPI, there was a significant increase in the percentage of IFN-γ^+^ CD4^+^ T cells compared to control animals.

### Maximal CD8^+^ T-Cell Response Requires Alveolar Macrophages but Not CD4^+^ T-Cells

**Figure 4:**
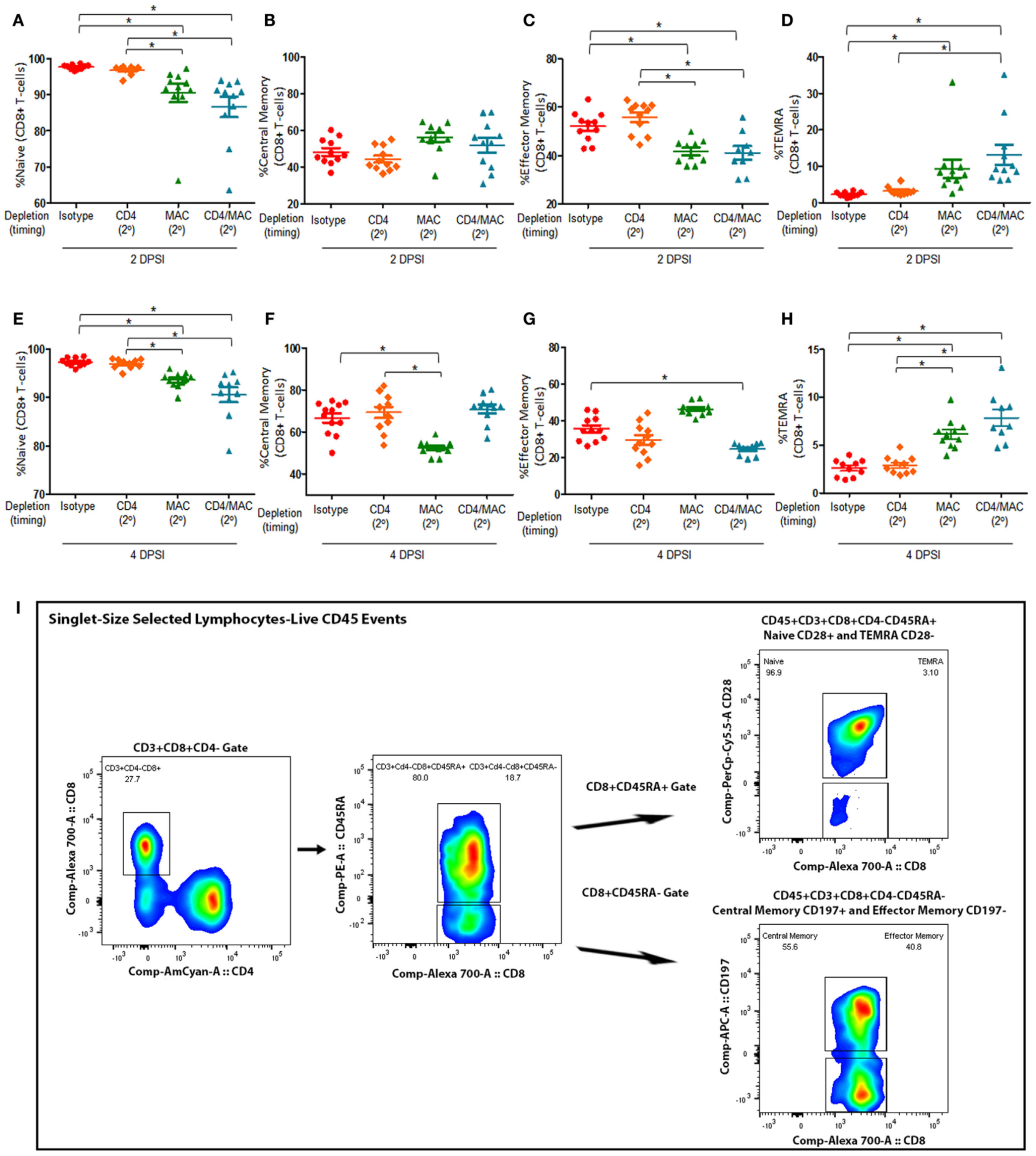
**Maximal CD8^+^ T-cell response require alveolar macrophages but not CD4**^+^ **T-cells**. Mice were infected intratracheally with *P. murina*, and groups of mice were depleted of CD4^+^ T-cells, macrophages, or CD4^+^ T-cells and macrophages every 6 days with depletion occurring prior to secondary infection (2°). Lung CD8^+^ T-cell subsets: naive CD8^+^ T cells at 2 and 4 days post-secondary infection [**(A,E)**, respectively], central memory CD8^+^ T cells at 2 and 4 DPI [**(B,F)**, respectively], effector memory CD8^+^ T cells at 2 and 4 DPI [**(C,G)**, respectively], and TEMRA CD8^+^ T cells at 2 and 4 DPI [**(D,H)**, respectively] were quantified *via* flow cytometry. **(I)** Representative gating strategy used for flow cytometry analysis. Dots and bars represent mean ± SEM, *N* = 10 at each time points, * indicates *P* < 0.05, for the indicated comparisons for each of the indicated time point, by Kruskal–Wallis ANOVA followed by Dunn’s posttest. DPSI, days post-secondary infection.

We then assessed the composition and quantity of various lung CD8^+^ T-cell subsets. We first examined the levels of naive CD8^+^ T (Figures 4A,E), central memory CD8^+^ T (Figures 4B,F), effector memory CD8^+^ T cells (Figures 4C,G), and terminally differentiated effector memory (TEMRA) CD8^+^ T cells (Figures 4D,H) in mice depleted of CD4^+^ T-cells, alveolar macrophages, or CD4^+^ T-cells and alveolar macrophages prior to a secondary infection at 2 and 4 days post-secondary infection. The gating strategy used to determine the various CD8^+^ T-cell phenotypes are depicted in Figure 4I. We defined the CD8^+^ T-cell subsets as naive (CD45^+^CD3^+^CD4^−^CD8^+^CD45RA^+^CD28^+^), central memory (CD45^+^CD3^+^CD4^−^CD8^+^CD45RA^−^CD197^+^), effector memory (CD45^+^CD3^+^CD4^−^CD8^+^CD45RA^−^CD197^−^), and TEMRA (CD45^+^CD3^+^CD4^−^CD8^+^CD45RA^+^CD28^−^). Interestingly, loss of CD4^+^ T-cells did not significantly affect the percentage of any of the CD8^+^ T-cell subsets, suggesting that CD8^+^ T-cell immune memory remains active despite loss of CD4^+^ T-cells. However, mice depleted of alveolar macrophages prior to a secondary infection exhibited a significant decrease in the percentage of effector memory CD8^+^ T-cells, as well as a significant increase in the percentage of TEMRA CD8^+^ T cells at both 2 and 4 DPI. We also found that mice depleted of macrophages and CD4^+^ T-cells prior to a secondary infection exhibited a significant decrease in the amount of naive CD8^+^ T-cells at 2 DPI, and this trend remained at 4 DPI. The percentage of central memory CD8^+^ T cells in the lung 2 DPI in immune-intact animals did not significantly differ from CD4-depleted animals or macrophage-depleted mice. However, by 4 DPI, the percentage of central memory CD8^+^ T cells significantly decreased in macrophage-depleted mice. These results suggest that loss of CD4^+^ T-cells does not affect the immune memory response of CD8^+^ T-cells, but that loss of macrophages or loss of both macrophages and CD4^+^ T-cells drives the CD8^+^ T-cell response toward a terminally differentiated effector memory phenotype.

### Loss of Alveolar Macrophages Increases IFN-γ Production by CD8+ T-Cells in Secondary Immune Responses to *Pneumocystis*

**Figure 5:**
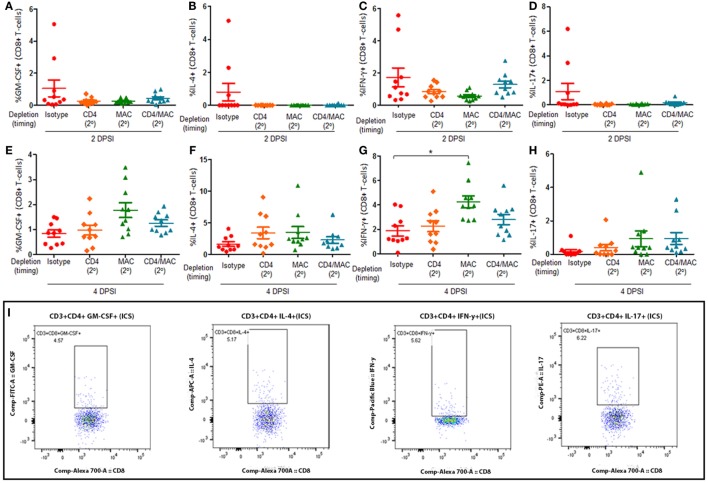
**Loss of alveolar macrophages increases IFN-γ production by CD8**^+^ **T-cells in secondary immune responses to *Pneumocystis***. C57BL/6 mice were infected with *P. murina*, and depleted of CD4^+^ T-cells, macrophages, or CD4^+^ T-cells and macrophages every 6 days with depletion occurring prior to secondary infection (2°). Lung CD8^+^ T-cell subsets: GM-CSF^+^ CD8^+^ T cells at 2 and 4 days post-secondary infection [**(A,E)**, respectively], IL-4^+^ CD8^+^ T cells at 2 and 4 DPI [**(B,F)**, respectively], IFN-γ^+^ CD8^+^ T cells at 2 and 4 DPI [**(C,G)**, respectively], and IL-17^+^ CD8^+^ T cells at 2 and 4 DPI [**(D,H)**, respectively] were quantified *via* flow cytometry. **(I)** Representative gating strategy used for flow cytometry analysis. Dots and bars represent mean ± SEM, *N* = 10 at each time points, * indicates *P* < 0.05, for the indicated comparisons for each of the indicated time point, by Kruskal–Wallis ANOVA followed by Dunn’s posttest. DPSI, days post-secondary infection.

Mice were infected with *P. murina* as described above, and groups of mice were depleted of CD4^+^ T-cells and macrophages every 6 days with depletion occurring prior to secondary infection (2°). Lung CD8^+^ T-cell subsets were then examined at 2 and 4 days post-secondary infection: GM-CSF^+^ CD8^+^ T cells (Figures 5A,E, respectively), IL-4^+^ CD8^+^ T cells (Figures 5B,F, respectively), IFN-γ^+^ CD8^+^ T cells (Figures 5C,G, respectively), and IL-17^+^ CD8^+^ T cells (Figures 5D,H, respectively). The gating strategy used to determine the various CD8^+^ T-cell phenotypes are depicted in Figure 5I. The percentage of GM-CSF^+^, IL-17^+^, or IL-4^+^ CD8^+^ T cells in the lung 2 or 4 DPI in immune-intact animals did not significantly differ from CD4-depleted animals or macrophage-depleted mice. However, loss of macrophages significantly increased the percentage of IFN-γ^+^ CD8^+^ T cells compared to control animals 4 DPI. These results suggest that loss of macrophages significantly increases the production of IFN-γ by CD8^+^ T-cells.

### Secondary Immune Responses Significantly Increase the Number of *P. murina*-Specific Bone Marrow Plasma Cells and *P. murina*-Specific IgG Enhances Macrophage Killing

**Figure 6:**
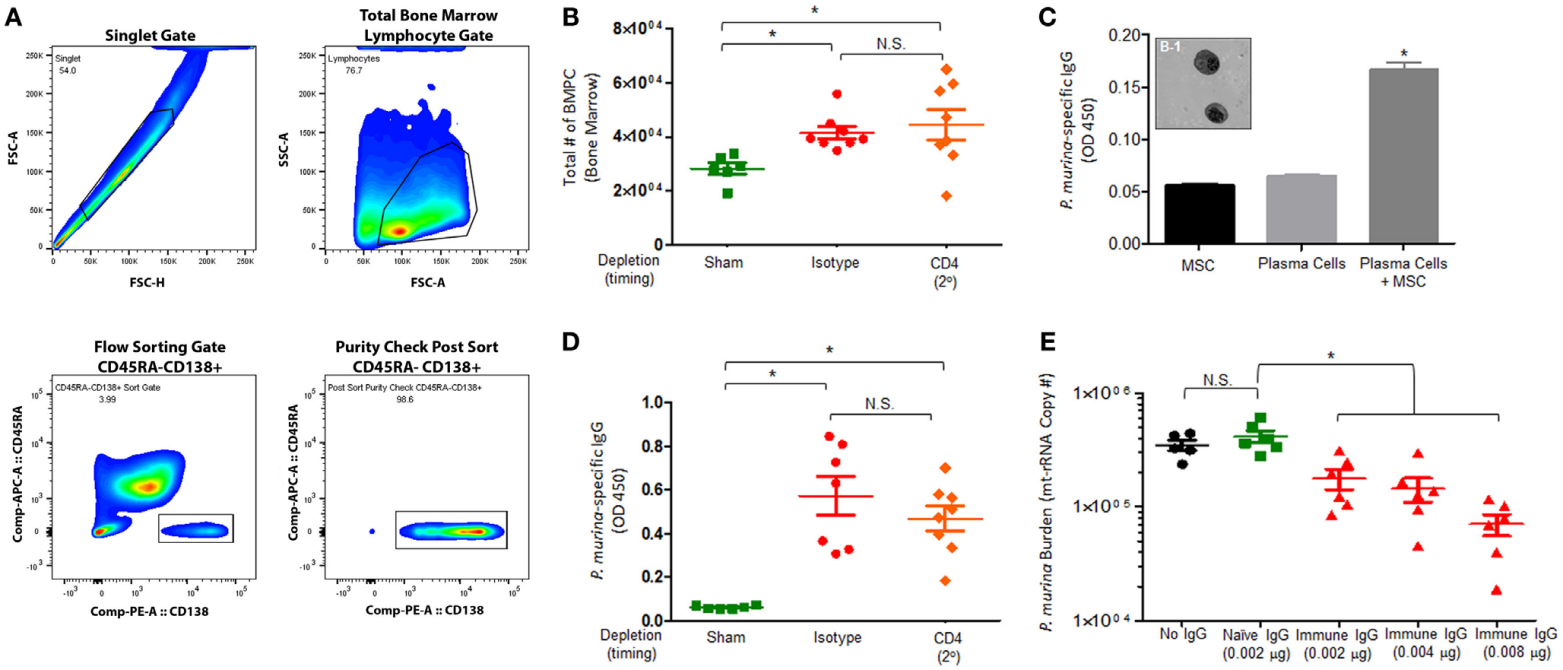
***Pneumocystis* pneumonia significantly increases the level of *P. murina*-specific bone marrow plasma cells and *P. murina*-specific IgG enhances macrophage killing**. C57BL/6 mice were infected with *P. murina* and depleted of CD4^+^ T-cells every 6 days with depletion occurring prior to secondary infection (2°). **(A)** Representative gating strategy used for flow cytometry analysis. **(B)** The absolute number of bone marrow plasma cell significantly increases in *P. murina* infected animals. **(C)** Bone marrow plasma cells isolated from infected animals secreted high levels of *P. murina*-specific IgG. **(C-1)** Representative micrograph of bone marrow plasma cells isolated from infected animals. **(D)** *P. murina*-specific IgG in the serum was assayed by ELISA. **(E)** *P. murina*-specific IgG enhanced *in vitro* killing of *P. murina* by macrophages in a dose-dependent manner. Dots and bars represent mean ± SEM, *N* = 10 at each time points, * indicates *P* < 0.05, for the indicated comparisons for each of the indicated time point, by Kruskal–Wallis ANOVA followed by Dunn’s posttest.

We next wanted to assess if infection with *Pneumocystis* generates long-lived bone marrow plasma cells and if *Pneumocystis*-specific antibodies enhance macrophage-mediated killing. Mice were infected with *P. murina* and depleted of CD4^+^ T-cells every 6 days with depletion occurring prior to secondary infection (2°), as described previously. The gating strategy used to flow sort BMPCs is described in Figure 6A. We first examined the absolute number of bone marrow plasma cells in *P. murina*-infected animals and found that there was a significant increase in the amount of obtainable BMPCs relative to sham-infected controls (Figure 6B). Additionally, loss of CD4^+^ T-cells prior to secondary infection did not significantly affect the number of BMPCs. To confirm that the BMPCs isolated from infected mice secreted *Pneumocystis*-specific IgG, BMPCs were grown in coculture with MSCs, and the levels of *P. murina*-specific IgG in the supernatant were determined. We found that the bone marrow plasma cells isolated from infected animals secreted high levels of *P. murina*-specific IgG (Figure 6C). A representative micrograph of bone marrow plasma cells isolated from infected animals is shown in Figure 6C and 1. We also found that the levels of *P. murina*-specific IgG in the serum was significantly increased in infected animals and was correlated with the increase in BMPCs (Figure 6D). Finally, we assessed the capacity of *P. murina*-specific IgG to enhance *in vitro* killing of *P. murina* by macrophages. We found that IgG from infected animals significantly increased the killing of *P. murina* by macrophages, in a dose-dependent manner (Figure 6E).

## Discussion

Exposure to *Pneumocystis* is ubiquitous in humans and occurs at an early age, around 2 years of life (2, 9). However, *Pneumocystis* pneumonia is a major cause of morbidity and mortality among immunocompromised patients, especially in the context of HIV/AIDS. This is surprising as the majority of adults have detectable levels of *Pneumocystis*-specific antibodies at the time of infection (10). These data suggest that immunological memory against *Pneumocystis* in humans may be insufficient or suboptimal, that other cellular populations (non-CD4^+^ T-cells) may be required for maximal secondary immune responses, or that HIV infection disrupts immune memory responses. Despite the fact that nearly all humans are exposed to *Pneumocystis*, there remains a paucity of data investigating the immunological memory and secondary immune response against *Pneumocystis*, especially in the context of CD4^+^ T-cell depletion. Therefore, we sought to investigate immunological memory against *Pneumocystis* in the context of CD4^+^ T-cell depletion.

Our current experiments demonstrate that secondary immune responses to *Pneumocystis* do not require CD4^+^ T-cells, which is consistent with the previous reports (3, 11, 12). However, we further went on to investigate other cellular populations that could be involved in secondary immune memory responses by investigating the role of CD8^+^ T-cells and macrophages during the immune memory recall response to *Pneumocystis*. We found that loss of CD8^+^ T-cells or macrophages alone or in the context of CD4^+^ T-cell depletion significantly impaired the ability of the host to clear *Pneumocystis* infection. We also evaluated the cellular phenotypes associated with CD4^+^ T-cells and CD8^+^ T-cells during secondary immune responses, in the context of CD4^+^ T-cell depletion, CD8^+^ T-cell depletion, and alveolar macrophage depletion. Loss of CD8^+^ T-cells or macrophages delayed the production of IFN-γ by CD4^+^ T-cells; however, by 4 DPI, the production is increased, which is likely due to increased fungal burden and the host trying to mediate a more robust immune response. While CD4^+^ T-cells are absolutely critical for resolution of *Pneumocystis* through the coordination of host inflammatory responses by recruitment and activation of effector cells, which ultimately lead to the elimination of the organism during a primary infection, loss of CD4^+^ T-cells does not render a previously exposed animal susceptible to re-infection with *Pneumocystis* (1).

Additionally, loss of CD4^+^ T-cells does not affect the immune memory response of CD8^+^ T-cells, but loss of macrophages or loss of both macrophages and CD4^+^ T-cells drives the CD8^+^ T-cell response toward terminally differentiated effector memory phenotype. Loss of macrophages also significantly increased the production of IFN-γ by CD8^+^ T-cells, which is likely due to increased fungal burden. Several studies have examined the role of CD8^+^ T-cells during primary infection, yet the role of CD8^+^ T-cells, particularly in the setting of CD4^+^ T-cell deficiency, remains controversial. Specifically, Gigliotti et al. demonstrated that there was no difference in organism burden in CD4^+^ T-cell depleted vs. CD4^+^ and CD8^+^ T-cell-depleted animals, indicating that CD8^+^ T cells are not involved in fungal clearance (13). However, other studies have shown that CD8^+^ T cells can be protective against *Pneumocystis* infection, though this is dependent on their cytotoxic phenotype, which is defined by high levels of endogenous IFN-γ production (14). In fact, animal studies have demonstrated that delivery of IFN-γ *via* aerosol or by an adenoviral-mediated gene transfer results in clearance of *Pneumocystis* in the absence of CD4^+^ T cells, and this was associated with an increase in recruited IFN-γ-producing CD8^+^ T cells (15). Further, McAllister et al. showed that IFN-γ stimulated CD8^+^ T cells from *Pneumocystis*-infected CD4-depleted mice enhanced macrophage-mediated *in vitro* killing (16). These studies suggest that the role of CD8^+^ T-cells during a primary infection is to enhance macrophage-mediated killing through the production of IFN-γ, which is consistent with our data assessing the role of CD8^+^ T-cells in secondary immune responses. More precisely, we observe loss of macrophages, but not CD4^+^ T-cells, significantly increases the production of IFN-γ by CD8^+^ T-cells and drives CD8^+^ T-cells toward a terminally differentiated effector memory phenotype [a cellular phenotype characterized by high levels of IFN-γ production (17)], all of which suggest that CD8^+^ T-cells upregulate IFN-γ responses to combat *Pneumocystis* infection during a secondary immune response. While we cannot fully explain these results, we believe that the increased fungal burden seen in macrophage-depleted mice results in increased activation of the remaining immune effector cells in the lung, including IFN-γ producing CD8^+^ T-cells.

Finally, we found that *Pneumocystis*-specific IgG from infected animals significantly increases the killing of *P. murina* by macrophages, in a dose-dependent manner. Alveolar macrophages are critical in optimal host defense against *Pneumocystis* as they have ability to directly kill both trophozoites and cysts (18, 19). Several studies have shown an inverse correlation between macrophage numbers and severity of *Pneumocystis* infection (20, 21). Specifically, animal studies demonstrate that chemical depletion of alveolar macrophages in normal rats, followed by *Pneumocystis* challenge results in significantly higher fungal load in the lungs compared with non-depleted mice (22). However, little data exist examining the role of macrophages during secondary immune responses to *Pneumocystis*. Macrophages utilize several mechanisms to kill *Pneumocystis* (i.e., nitric oxide release and opsonization) (23). More precisely, alveolar macrophages primed by IFN-γ produce a nitrosative burst that is toxic to *Pneumocystis* (23). Additionally, Roths and Sidman found that anti-*Pneumocystis* serum was effective at reducing the number of organisms in all stages of *Pneumocystis* infection in SCID mice (24). Taken together, these studies suggest that macrophage-mediated killing of *Pneumocystis* is mediated, in part, by IFN-γ stimulation and opsonization *via Pneumocystis*-specific IgG, which is consistent with our data that demonstrate that *Pneumocystis*-specific IgG from infected animals significantly increases the killing of *P. murina* by macrophages, and that loss of macrophages significantly increases the production of IFN-γ by CD8^+^ T-cells.

The host immune memory response during *Pneumocystis* infection involves a complex interaction between CD4^+^ T-cells, CD8^+^ T-cells, alveolar macrophages, and soluble factors that together facilitate clearance of the infection. Our data suggest that the immune memory response to *Pneumocystis* is, in part, mediated by CD8^+^ T-cells, macrophages, and *Pneumocystis*-specific IgG, but that CD4^+^ T-cells play little role. In addition, infection with *Pneumocystis* significantly increases the levels of *Pneumocystis*-specific long-lived BMPCs, and numbers of BMPCs are not significantly affected by loss of CD4^+^ T-cells. Taken together, these results suggest that investigation of immune cell populations other than CD4^+^ T-cells during immune responses to *Pneumocystis* in the HIV^+^ population may be extremely valuable.

## Ethics Statement

The study was approved by the LSUHSC Institutional Animal Care and Use Committee.

## Author Contributions

Conception and design: DS, TC, NR, DW, and JS; analysis and interpretation: DS, TC, NR, JS, and DW; drafting the manuscript: DS and NR.

## Conflict of Interest Statement

The authors declare that the research was conducted in the absence of any commercial or financial relationships that could be construed as a potential conflict of interest.
